# Interactions between decision-making and emotion in behavioral-variant frontotemporal dementia and Alzheimer’s disease

**DOI:** 10.1093/scan/nsaa085

**Published:** 2020-07-01

**Authors:** Aurélie L Manuel, Daniel Roquet, Ramon Landin-Romero, Fiona Kumfor, Rebekah M Ahmed, John R Hodges, Olivier Piguet

**Affiliations:** 1 School of Psychology, The University of Sydney, Sydney, Australia; 2 Brain & Mind Centre, The University of Sydney, Sydney, Australia; 3 ARC Centre of Excellence in Cognition & its Disorders, Sydney, Australia; 4 Laboratory for Research in Neuroimaging LREN, Department of Clinical Neurosciences, Lausanne University Hospital and University of Lausanne, Lausanne, Switzerland; 5 Clinical Medical School, The University of Sydney, Sydney, Australia

**Keywords:** delay discounting, emotion, ventromedial prefrontal cortex, amygdala, voxel-based morphometry

## Abstract

Negative and positive emotions are known to shape decision-making toward more or less impulsive responses, respectively. Decision-making and emotion processing are underpinned by shared brain regions including the ventromedial prefrontal cortex (vmPFC) and the amygdala. How these processes interact at the behavioral and brain levels is still unclear. We used a lesion model to address this question. Study participants included individuals diagnosed with behavioral-variant frontotemporal dementia (bvFTD, *n* = 18), who typically present deficits in decision-making/emotion processing and atrophy of the vmPFC, individuals with Alzheimer’s disease (AD, *n* = 12) who present with atrophy in limbic structures and age-matched healthy controls (CTRL, *n* = 15). Prior to each choice on the delay discounting task participants were cued with a positive, negative or neutral picture and asked to vividly imagine witnessing the event. As hypothesized, our findings showed that bvFTD patients were more impulsive than AD patients and CTRL and did not show any emotion-related modulation of delay discounting rate. In contrast, AD patients showed increased impulsivity when primed by negative emotion. This increased impulsivity was associated with reduced integrity of bilateral amygdala in AD but not in bvFTD. Altogether, our results indicate that decision-making and emotion interact at the level of the amygdala supporting findings from animal studies.

## Introduction

Emotions play an important part in many of our decisions (Bechara *et al.*, 2000; Clore and Huntsinger, 2007). Choosing to save for our children’s education rather than buying our dream car not only involves options with different reward magnitude and delays but also options with distinctive affective content. How emotions interact with decision-making processes, however, is still largely unresolved.

The well-established delay discounting task measures the ability to forgo immediate small rewards in favor of larger longer-term rewards (Green and Myerson, 2004). Choosing larger-later rewards over smaller-sooner rewards (e.g. $70 in 40 days over $50 now) is associated with enhanced performance across the lifespan including better academic performance, social relationships and more adaptive social functioning (Hirsch *et al.*, 2008; Shamosh and Gray, 2008). In contrast, the tendency to choose the smaller-sooner rewards over larger-later rewards has been associated with impulsivity-related behaviors and pathological conditions including drug dependence (Bickel and Marsch, 2001), gambling (Reynolds, 2006) or eating disorders (Kekic *et al.*, 2016). Neuroimaging studies investigating delay discounting consistently point to a core network including the ventromedial prefrontal cortex (vmPFC), amygdala, anterior cingulate cortex (ACC) and striatum (McClure *et al.*, 2004; Kable and Glimcher, 2007; McClure *et al.*, 2007; Ballard and Knutson, 2009; Peters and Buchel, 2009). Studies, however, report inconsistent findings, possibly due to focused region-of-interest analyses (Kable and Levy, 2015) or healthy populations where no structural abnormalities are reported (Bjork *et al.*, 2009; Bernhardt *et al.*, 2014; Tschernegg *et al.*, 2015). Greater delay discounting has been associated with reduced grey matter intensity in striatum (Dombrovski *et al.*, 2012; Cho *et al.*, 2013), vmPFC (Bernhardt *et al.*, 2014; Pehlivanova *et al.*, 2018), lateral prefrontal cortex (Bjork *et al.*, 2009), superior frontal gyrus (Schwartz *et al.*, 2010), ACC (Bernhardt *et al.*, 2014), hippocampus (Lebreton *et al.*, 2013), insula (Turel *et al.*, 2018) as well as temporal pole and temporoparietal junction (Pehlivanova *et al.*, 2018). Conversely, greater delay discounting has also been associated with increased grey matter intensity of the striatum (Schwartz *et al.*, 2010; Tschernegg *et al.*, 2015), vmPFC and ACC (Cho *et al.*, 2013) and prefrontal cortex (Wang *et al.*, 2016). Lesion studies have shown that vmPFC lesion increases delay discounting (i.e. impulsivity), compared with healthy controls or with individuals with lesions in other brain regions (Sellitto *et al.*, 2011; Peters and D'Esposito, 2016). In rodents, lesions of the basolateral amygdala (BLA) (Winstanley *et al.*, 2004; Floresco and Ghods-Sharifi, 2007; Ghods-Sharifi *et al.*, 2009) or disconnection between the orbitofrontal cortex (OFC) and the BLA (Churchwell *et al.*, 2009) increases delay discounting similar to lesions to the OFC (Mobini *et al.*, 2002). To our knowledge, no studies have investigated the effect of amygdala damage on delay discounting in humans. Evidence shows that patients with focal amygdala damage have reduced loss aversion (De Martino *et al.*, 2010) and lower scores on tasks of decision-making under risk (Bechara *et al.*, 1999; Bar-On *et al.*, 2003; Hanten *et al.*, 2006; Brand *et al.*, 2007; Weller *et al.*, 2007) or under ambiguity conditions (Brand *et al.*, 2007).

Relevant to this study, some key regions underlying decision-making—vmPFC and amygdala—are known to play a central role in emotion processing (Hommer *et al.*, 2003; Lindquist *et al.*, 2012; Herman *et al.*, 2018; Kelley *et al.*, 2018) and are extensively connected (Haber and Knutson, 2010; Schardt *et al.*, 2010; Patin and Hurlemann, 2011; Plichta and Scheres, 2014). While the vmPFC appears to respond to both negative and positive stimuli (Winecoff *et al.*, 2013; Yang *et al.*, 2020), the amygdala is traditionally known from animal and human lesion studies as the hub for processing negative emotions (LeDoux, 1998; Adolphs *et al.*, 2005). Human neuroimaging studies also support the view for a central role of the amygdala in processing negative emotions (Davis and Whalen, 2001), although amygdala activation during positive emotion processing has been reported as well (Garavan *et al.*, 2001; Hamann and Mao, 2002). Because of their mutual connections, it is not surprising that contextual information such as emotion shifts choices on the delay discounting task toward being more patient or impulsive (Lempert and Phelps, 2016).

The majority of studies show that short (1.5 seconds, Guan *et al.*, 2015) or long (15 seconds, Augustine and Larsen, 2011) exposure to negative emotional pictures increases the propensity to choose smaller-sooner over larger-later rewards, whereas exposure to positive pictures shifts decisions toward choosing larger-later rewards (Guan *et al.*, 2015; Cai *et al.*, 2019). Similar findings were also reported in studies using emotional episodic future thinking as the emotional cue (Liu *et al.*, 2013; Lin and Epstein, 2014; Zhang *et al.*, 2018). Some studies find opposite findings, with effects specific to particular conditions, namely reports of increased delay discounting following positive emotion in extraverted individuals (Hirsh *et al.*, 2010) and decreased delay discounting following fearful faces (Luo *et al.*, 2014). Arousing pictures, regardless of emotion, also tend to increase delay discounting (Wilson and Daly, 2004; Sohn *et al.*, 2015).

This study aimed to identify the relations between decision-making and emotion processing and their biological mechanisms, using a lesion model. Inclusion of patients with behavioral-variant frontotemporal dementia (bvFTD) and Alzheimer’s disease (AD), presenting with atrophy in the key brain regions of the reward and emotion network (vmPFC, limbic lobe) will clarify the role of emotion on delay discounting and the contribution of each brain region in delay discounting. bvFTD is a neurodegenerative condition characterized by marked changes to personality and interpersonal conduct (Piguet *et al.*, 2017) as evidenced by their increase in ‘impulsive, rash or careless actions’ (Rascovsky *et al.*, 2011). Patients with bvFTD also show disruption in emotional processing (Lavenu *et al.*, 1999; Keane *et al.*, 2002; Fernandez-Duque and Black, 2005; Kipps *et al.*, 2009; Kumfor *et al.*, 2013a, 2014a). Atrophy is typically reported in emotion-specific brain regions namely in vmPFC and insula (Seeley *et al.*, 2008), which extends into subcortical regions with disease progression (Landin-Romero *et al.*, 2017). Given their behavioral deficits—decision-making and emotion processing—and atrophy of the vmPFC, we would anticipate a correlation between reduced grey matter intensity in the vmPFC and increased delay discouting, regardless of emotion.

The predominant clinical feature of Alzheimer’s disease in contrast is an impairment in episodic memory (McKhann *et al.*, 2011), mainly attributed to atrophy of structures of the medio-temporal limbic system such as hippocampus and amygdala (Scheltens *et al.*, 1992; McKhann *et al.*, 2011; Poulin *et al.*, 2011) and progressing to parietal, posterior cingulate and frontal cortices with disease (Nestor *et al.*, 2003; Dickerson *et al.*, 2009; Landin-Romero *et al.*, 2017). Early in the disease process, interpersonal behavior and emotion processing are relatively preserved in AD, although some facets of emotion processing and behavior are impaired (Cummings, 1997; Hoefer *et al.*, 2008) and worsen with disease progression (Bidzan *et al.*, 2012; Kumfor *et al.*, 2014b; Bertoux *et al.*, 2015a). AD patients, although overall capable of recognizing emotions, can be severely impaired in retrieving emotions relevant to autobiographical memories for example (Irish *et al.*, 2011; Kumfor *et al.*, 2013a). While emotion processing deficit is considered a core feature of bvFTD (Rascovsky *et al.*, 2011), emotion processing—to some extent—remains comparatively preserved in AD (Lavenu *et al.*, 1999). Despite relatively preserved decision-making and emotion processing compared to bvFTD, we would anticipate emotion to interact with delay discounting performance in AD. We would also expect reduced amygdalar grey matter integrity to increase delay discounting and weaken the interactions between emotions and decision-making.

Few studies have investigated delay discounting in bvFTD and AD. Increased delay discounting has been reported in bvFTD compared to AD (Lebreton *et al.*, 2013; Bertoux *et al.*, 2015b) and in healthy controls (Beagle *et al.*, 2020), while Chiong et al. (2016) reported similar performance between bvFTD, AD and controls. AD patients show a trend for increased delay discounting compared to healthy controls (Lebreton *et al.*, 2013; Bertoux *et al.*, 2015b; Beagle *et al.*, 2020). Brain-behavior associations with delay discounting performance in bvFTD and AD are less clear as most studies only included behavioral data (Bertoux *et al.*, 2015b), only reported patterns of brain atrophy (Lebreton *et al.*, 2013) or investigated brain-behavior correlations across etiologies (Lansdall *et al.*, 2017; Beagle *et al.*, 2020). The only study investigating brain-behavior correlations in bvFTD and AD (Chiong *et al.*, 2016) failed to find significant correlations between brain atrophy and delay discounting probably because of the lack of between-group behavioral differences. Only one study investigated or reported brain-behavior correlations in bvFTD and AD in decision-making tasks other than the delay discounting task (Kloeters *et al.*, 2013). Using the Iowa Gambling Task, this study found that decision-making deficits were attributed to frontal atrophy in bvFTD and to temporal/parietal atrophy in AD.

To identify the influence of emotion on delay discounting, we presented individuals diagnosed with bvFTD or AD, and healthy controls, emotional or neutral pictures before each choice on a delay discounting task. Given their divergent patterns of brain atrophy and clinical features, we predicted that bvFTD would exhibit greater impulsivity overall compared with the other two groups, and that AD would be more impulsive than controls. In addition, we hypothesized that due to their deficits in emotion processing, bvFTD would not show any emotion-induced modulation of delay discounting. In contrast, we expected AD to show a similar emotion-induced modulation of delay discounting than controls, namely increased delay discounting, that is impulsivity, for negative emotions and decreased delay discounting for positive emotions. At the anatomical level, we expected the decision-making deficits to relate to distinct neural structures (Kloeters *et al.*, 2013). Based on lesion studies (Sellitto *et al.*, 2011; Peters and D'Esposito, 2016), we predicted that increased delay discounting in the bvFTD group would correlate with decreased grey matter intensity in the vmPFC, regardless of emotional valence. In the AD group, given the limited vmPFC atrophy, we anticipated that atrophy of the amygdala and other limbic structures would be related to increased delay discounting as demonstrated in animal studies (Winstanley *et al.*, 2004; Floresco and Ghods-Sharifi, 2007; Ghods-Sharifi *et al.*, 2009). In addition, because of its central role in processing negative emotion, we also hypothesized that reduced grey matter intensity in the amygdala in AD would counteract the expected increased delay discounting in the negative condition.

## Methods

### Participants

Twenty-two patients diagnosed with bvFTD, 15 patients with AD and 15 education- and age-matched healthy controls were recruited from FRONTIER, the frontotemporal dementia research clinic in Sydney, Australia. Calculation of sample size was based on an a priori power analysis using G^*^Power (Faul *et al.*, 2007). For an alpha level of 0.05, an anticipated effect size of 0.06 (medium) and a power of 0.80, the estimated total sample is 36 participants (12 in each group). All patients underwent a comprehensive neurological examination, a neuropsychological assessment, and a structural brain MRI. Diagnosis was established according to relevant clinical diagnostic criteria at the time of testing for probable or possible bvFTD (Rascovsky *et al.*, 2011) and AD (McKhann *et al.*, 2011). Diagnosis was established by multidisciplinary agreement based on cognitive, clinical and imaging data. Exclusion criteria for patients and controls included: presence of a primary psychiatric disorder, presence of other dementia or neurological disorders, and/or history of alcohol or substance abuse. All healthy controls underwent the comprehensive neuropsychological assessment and the brain MRI and were required to score >88/100 on the ACE-III to ensure they did not have any significant cognitive impairments. All participants or their Person Responsible provided informed consent in accordance with the Declaration of Helsinki. The South Eastern Sydney Local Health District and the University of New South Wales ethics committees approved the study.

### Neuropsychological assessment

The ACE-III was used to assess general cognition (Hsieh *et al.*, 2013; So *et al.*, 2018). Disease severity was assessed with the Frontotemporal Lobar Degeneration-Modified Clinical Dementia Rating Scale Sums of Boxes (CDR-FTLD SoB) (Knopman *et al.*, 2008), and disease duration was measured in years from the first onset of symptoms.

### Delay discounting task

**Fig. 1:**
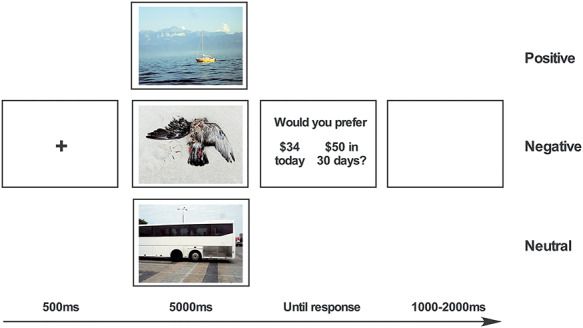
Experimental design. The delay discounting task consisted of three blocks containing either positive (POS), negative (NEG) or neutral (NEU) pictures and presented in randomized order. Participants were first instructed to vividly imagine witnessing the picture and then asked to make a choice on the delay discounting task.

The ability to delay gratification was assessed with the Monetary Choice Questionnaire (MCQ, Kirby *et al.*, 1999). The MCQ comprises 27 dichotomous choices asking participants to choose between a smaller, immediate monetary reward or a larger, delayed monetary reward (e.g. ‘Would you prefer $15 today or $35 in 13 days?’). Estimates of delay discounting were calculated for all reward magnitudes as well as for each different reward magnitude, categorized as low- ($25–35), medium- ($50–60) and high-magnitude ($75–85) trials. Indifference points were calculated with the classically used hyperbolic discounting equation: V=A/(1+*k*D) (Mazur, 1987) where V represents the present value of the delayed reward A at delay D, and *k* is a free parameter that determines the discount rate. Larger values for *k* indicate a preference for smaller immediate reward. Because of skewness, *k* values were log-transformed (log*k*) (Gray *et al.*, 2016). Although the monetary rewards were hypothetical, real and hypothetical rewards lead to similar patterns of discounting (Johnson and Bickel, 2002; Madden *et al.*, 2003). Prior to each choice, an emotional picture (Positive, POS; Negative, NEG; or Neutral, NEU) was presented for 5 seconds and participants were instructed to vividly imagine that they were witnessing the event/content depicted in it (Figure 1).

To control that they understood the task correctly, participants completed a training session consisting of three trials, during which they were asked on one random trial to indicate (i) which choice would pay sooner and (ii) which choice would pay greater. Only participants completing the training session and answering correctly the control questions were retained for the analyses.

Participants completed three blocks (POS, NEG or NEU) of the delay discounting task in a randomized order. Each trial began with a fixation cross presented on a 21.5 inch monitor for 500 ms, a picture displayed for 5000 ms and a screen containing both choices displayed until participants responded. An inter-stimulus interval (ISI) of 1000–2000 ms preceded the following trial. Participants indicated their choices by pressing the left or right arrow of a keyboard, according to the choice displayed on the left or the right of the screen. Each block lasted approximately 5 min. The three blocks were separated by a 5-minute break during which participants completed various questionnaires. Stimulus delivery and subjects' responses for both tasks were controlled using E-prime 2.0 software (Psychology Software Tools, Pennsylvania, USA).

The pictures were realistic, high-quality photographs chosen from the Nencki Affective Picture System (NAPS, Marchewka *et al.*, 2014). Pictures were selected on the basis of their original valence rating (1 = very negative, 5 = neutral, 9 = very positive) and ultimately designated as (mean ± standard deviation) positive (7.9 ± 0.2), negative (2.5 ± 0.3) or neutral (5.1 ± 0.2; F_(2,80)_ = 2628.93, *P* < 0.01). Arousal ratings also differed between positive (4.1 ± 0.1), negative (6.7 ± 0.5) and neutral pictures (4.8 ± 0.4; F_(2,80)_ = 105.97, *P* < 0.01). Stimuli were matched with respect to their luminance (F_(2,80)_ = 0.63, *P* = 0.53), contrast (F_(2,80)_ = 2.01, *P* = 0.14) and entropy (F_(2,80)_ = 2.02, *P* = 0.14).

### Questionnaires

Between each delay discounting block, participants completed the present and future sections of the Zimbardo Time Perspective Inventory, which comprises 37 items ranging from 1 (very untrue) to 5 (very true) and grouped into present-hedonistic, present-fatalistic and future dimensions (Zimbardo and Boyd, 2015).

At the end of the experimental session, participants rated valence and arousal for a subset of pictures (*n* = 15) of each emotion category using the Self-Assessment Manikin (Lang *et al.*, 1997) and a scale from 1 to 9 (valence: 1 = very negative to 9 = very positive; arousal: 1 = relaxed to 9 = aroused). The picture remained on the screen until the response was recorded.

### Statistical analyses

Data were analysed using IBM SPSS Statistics, 24.0 (SPSS Inc., Chicago, Ill., USA). Normally distributed variables, as determined with Shapiro–Wilks tests, were compared across groups using mixed or one-way ANOVAs followed by Sidak post hoc tests. Variables not normally distributed across our sample were analysed by Kruskal–Wallis ANOVA followed by Mann–Whitney U tests. Categorical measures (e.g. sex) were analysed by Chi-square tests. Effect sizes are reported using the partial eta-square (η^2^).

We investigated delay discounting (log*k*) with a 3 × 3 mixed ANOVA with within factor of Emotion (POS, NEG or NEU) and between factor of Group (bvFTD, AD and CTRL). Significant interactions were followed by simple effects at each combination of levels of the other factors and followed by Sidak post hoc tests. Additionnally, we investigated effects of Emotion for each reward magnitude separately using the same statistical analysis.

Correlations between the significant delay discounting conditions (Pos, Neg and Neu) in bvFTD and AD and respective valence/arousal ratings (Pos, Neg and Neu) were analysed using Spearman rank coefficient. Only correlations surviving Bonferroni correction for multiple comparisons were kept.

### Neuroimaging analyses

#### MRI acquisition

Participants underwent whole-brain structural MRI on a GE Discovery MR750 3T scanner equipped with an 8-channel head coil. High resolution 3D BRAVO T1-weighted images were acquired using the following parameters: imaging matrix of 256 × 256 × 200, 1 mm isotropic voxel resolution, echo time = 2.5 ms, repetition time = 6.7 ms, inversion time = 900 ms, flip angle = 8°.

#### Data pre-processing

Voxel-based morphometry (VBM) was conducted using SPM12 (Welcome Department of Cognitive Neurology, London, UK), in Matlab R2018a (Mathworks, Natick, Massachusetts, USA). First, T1-weighted images were segmented into six tissue probability maps in the native space. Both the original T1-weighted and the segmented maps were screened during an image quality control. Two participants (1 bvFTD and 1 AD) were removed for the subsequent pre-processing steps and statistical analyses due to motion during the acquisition or segmentation failure. A DARTEL template was computed using all the grey and white matter probability maps which satisfied our criteria for quality control. Last, grey matter probability maps were spatially normalized to the Montreal National Institute (MNI) space according to the transformation parameters from the corresponding DARTEL template. Images were modulated and smoothed with a Gaussian filter of full width at half maximum of 8 mm.

#### VBM analyses

Patterns of grey matter intensity decrease were explored using a whole-brain general linear model comprising bvFTD, AD and CTRL groups as well as age and total intracranial volume (to account for individual differences in head size) as regressors of non-interest. The total intracranial volume was assessed in the patient’s space prior to spatial normalization by summing thresholded grey matter, white matter and corticospinal fluid probability maps (threshold = 0.2) and counting non-zero voxels. Differences in grey matter intensities between groups (bvFTD *vs* control; AD *vs* control) were assessed using t-tests.

Next, correlations between delay discounting and grey matter intensity were investigated. Scores for each delay discounting condition (POS, NEG or NEU) were entered simultaneously into the design matrix. Age and total intracranial volume were included as regressors of non-interest. Correlations were first investigated between delay discounting and grey matter intensity combining all participants (bvFTD, AD and CTRL). Then, the same analyses described above were conducted to investigate correlations in each patient group combined with controls in order to identify the neural correlates of delay discounting distinct to each patient group. Inclusion of controls has been shown to increase statistical power to detect brain–behavior relationships across the entire brain (e.g. Kumfor *et al.*, 2013b).

Voxel-wise statistical analyses are reported using a cluster size of at least 50 voxels, at statistical threshold of *P* < 0.001, uncorrected for multiple comparison. This approach minimizes Type I error while balancing the risk of Type II error (Lieberman and Cunningham, 2009). Significant results were overlaid on the Montreal Neurological Institute (MNI) standard brain using MRIcron (https://www.nitrc.org/projects/mricron).

## Results

### Demographic and neuropsychological profiles

Twenty-two individuals diagnosed with bvFTD, 15 with Alzheimer’s disease and 15 older healthy controls were recruited for this study. Seven participants (4 bvFTD and 3 AD), however, failed the delay discounting task training session, and their data were therefore removed from the analyses. As such, the final samples included 18 bvFTD, 12 AD and 15 CTRL participants. As reported in Table 1, groups were well matched on age (*P* = 0.636). Although groups are statistically matched on sex (*P* = 0.080) and education level (*P* = 0.066), the bvFTD group is marginally composed of more men of lower education than the control or AD groups. Patient groups did not differ on disease duration (*P* = 0.261) or disease severity (CDR-FTLD Sob, *P* = 0.989) either. AD patients were however significantly more impaired on general cognition than bvFTD (ACE-III, *P* < 0.001). Both patient groups had significantly greater disease severity (*P* < 0.001) and impaired general cognition (*P* < 0.001) than controls. Excluded participants tended to be more impaired on the ACE-III than their respective samples (bvFTD included: 82.0 ± 9.9, bvFTD excluded: 73.2 ± 14.6, *P* = 0.15; AD included: 65.9 ± 12.6, AD excluded: 53.6 ± 6.8, *P* = 0.06).

**Table 1:** Demographic and clinical information

	bvFTD (*n* = 18)	AD (*n* = 12)	CTRL (*n* = 15)	*P*	Post hoc
**Sex (M:F)**	13:5	7:5	5:10	0.080^*^	
**Age**	63.4 ± 9.8	65.5 ± 7.3	65.7 ± 4.7	0.636	
**Education (yrs)**	11.7 ± 3.0	13.6 ± 3.6	13.9 ± 2.7	0.066^#^	
**Disease duration (yrs)**	8.3 ± 5.9	5.1 ± 3.1	−	0.261^†^	
**ACE-III (/100)**	82.0 ± 9.9	65.9 ± 12.6	94.8 ± 3.0	< 0.001	Patients < Controls; AD < bvFTD
**CDR-FTLD Sob**	5.3 ± 4.6	5.0 ± 3.5	0.2 ± 0.2	< 0.001	Patients < Controls

Demographic and clinical information for behavioral-variant frontotemporal dementia (bvFTD), Alzheimer’s disease (AD) and controls (CTRL). Values are mean ± standard deviation. ^*^χ2 test, †Mann–Whitney, #Kruskal–Wallis test. ACE-III = Addenbrooke’s Cognitive Examination—Third edition; CDR-FTLD SoB = Frontotemporal Lobar Degeneration-Modified Clinical Dementia Rating Scale Sums of Boxes (CDR-FTLD SoB). Missing Scores: Education (2 AD); Disease duration (4 AD); ACE-III (1 AD), CDR-FTLD SoB (1 AD, 3 CTRL).

### Delay discounting results

**Figure 2:**
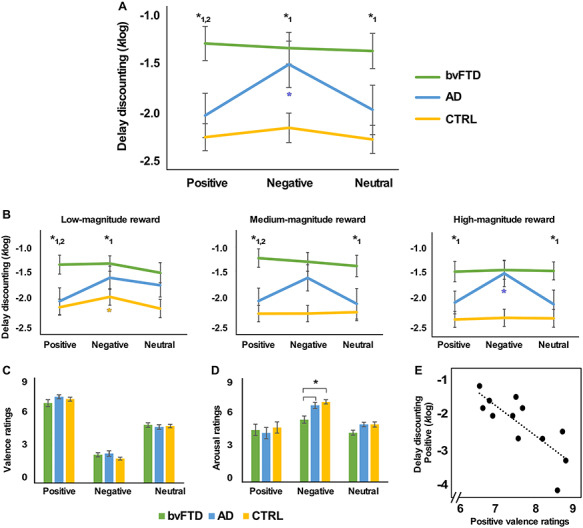
Behavioral results and correlations. A. Average delay discounting rates (*k*, log transformed) for each Emotion condition (Positive, Negative and Neutral) and Group (behavioral-variant frontotemporal dementia, bvFTD; Alzheimer’s disease, AD; controls, CTRL). B. Delay-discounting rate for low-, medium- and high-magnitude rewards. C, D. Judgement of valence and arousal for each Emotion condition and Group. E. Correlation between delay discounting in the Positive condition and positive valence ratings in the AD group. Graph lines and bars show means and standard error of the mean. ^*^ indicates significant post hoc differences (*P* < 0.05, Sidak corrected for multiple comparisons) for bvFTD < AD, CTRL (^*^1) and bvFTD = AD < CTRL (^*^2). Colored ^*^ indicates effects of Emotion in each Group.

Performance on the delay discounting task is illustrated in Figure 2A (and Supplementary Figure 2, which displays individual delay discounting rate for each condition). The ANOVA on delay discounting rate (log*k*) revealed a significant main effect of Emotion (F_(2,84)_ = 6.316, *P* = 0.003, η_p_^2^ = 0.131), Group (F_(2,42)_ = 7.504, *P* = 0.002, η_p_^2^ = 0.263) and an Emotion x Group interaction (F_(4,84)_ = 3.669, *P* = 0.008, η_p_^2^ = 0.149). Simple effects tests for each level of factor Group showed a main effect of Emotion for AD (F_(2,22)_ = 5.717, *P* = 0.010, η_p_^2^ = 0.342) but not for bvFTD (F_(2,34)_ = 0.306, *P* = 0.739, η_p_^2^ = 0.018) or CTRL (F_(2,28)_ = 2.116, *P* = 0.139, η_p_^2^ = 0.131). AD showed increased delay discounting (i.e. impulsivity) in the NEG compared to POS (*P* = 0.003) and NEU conditions (*P* = 0.036). Simple effects tests for each level of factor Emotion showed main effects of Group for all 3 emotions (all *P* < 0.01). Patients with bvFTD patients were significantly more impulsive than CTRL on all emotion conditions (all *P* < 0.002) and significantly more impulsive than AD on POS (*P* = 0.007) and NEU conditions (*P* = 0.033). AD patients were significantly more impulsive than CTRL on the NEG condition only (*P* = 0.024).

Figure 2B displays delay discounting rates for each reward magnitude. For low-magnitude rewards, significant main effects of Emotion (F_(2,84)_ = 3.389, *P* = 0.038, η_p_^2^ = 0.075) and Group (F_(2,42)_ = 5.133, *P* = 0.010, η_p_^2^ = 0.196) were present but no Emotion x Group interaction (F_(4,84)_ = 1.358, *P* = 0.256, η_p_^2^ = 0.061). In other words, participants were more impulsive in the NEG than POS (*P* = 0.011) or NEU (*P* = 0.030) conditions and the bvFTD group was more impulsive than the CTRL group (*P* = 0.003). Focused analyses on the control group showed that the CTRL were more impulsive in the NEG compared to POS condition (*P* = 0.010).

For medium-magnitude rewards, a significant main effect of Group was observed (F_(2,42)_ = 8.359, *P* = 0.001, η_p_^2^ = 0.285) but not of Emotion (F_(2,84)_ = 2.232, *P* = 0.114, η_p_^2^ = 0.050) or Emotion x Reward interaction (F_(4,84)_ = 2.118, *P* = 0.086, η_p_^2^ = 0.092). The bvFTD group was more impulsive than AD (*p* = 0.022) and CTRL (*P* < 0.001).

For high-magnitude rewards, significant main effects of Emotion (F_(2,84)_ = 5.142, *P* = 0.008, η_p_^2^ = 0.109), Group (F_(2,42)_ = 7.061, *P* = 0.002, η_p_^2^ = 0.252), as well as an Emotion x Group interaction (F_(4,84)_ = 3.694, *P* = 0.008, η_p_^2^ = 0.150) were present. AD patients were more impulsive in the NEG compared to POS (*P* < 0.001) and NEU (*P* = 0.014) conditions. The bvFTD group was more impulsive than CTRL in all conditions (*P* < 0.05) and than AD in POS (*P* = 0.036) and NEU (*P* = 0.031) conditions.

Regarding difference between reward magnitudes, main effects were present in the CTRL (F_(2,28)_ = 6.228, *P* = 0.006, η_p_^2^ = 0.308) but not in bvFTD (F_(2,34)_ = 1.589, *P* = 0.219, η_p_^2^ = 0.085) or AD groups (F_(2,22)_ = 0.332, *P* = 0.721, η_p_^2^ = 0.029). CTRL were more impulsive on low-magnitude trials than medium- (*P* = 0.011) or high-magnitude trials (*P* = 0.016).

### Questionnaires

Regarding valence ratings, a significant main effect of Emotion (F_(2,84)_ = 419.367, *P* < 0.001, η_p_^2^ = 0.909) was observed but not of Group (F_(2,42)_ = 0.598, *P* = 0.555, η_p_^2^ = 0.028) or an Emotion x Group interaction (F_(4,84)_ = 1.324, *P* = 0.268, η_p_^2^ = 0.059). Valence ratings differed significantly between positive, negative and neutral pictures for all groups (*P* < 0.001; Figure 2C and D).

Regarding arousal ratings, significant main effects of Emotion (F_(2,84)_ = 26.278, *P* < 0.001, η_p_^2^ = 0.385) and Group (F_(2,42)_ = 3.892, *P* = 0.028, η_p_^2^ = 0.156) were observed but no interaction (F_(4,84)_ = 1.753, *P* = 0.146, η_p_^2^ = 0.077). Negative pictures were judged as more arousing compared to positive and neutral pictures (all *P* < 0.001) and patients with bvFTD judged pictures as less arousing/more relaxing than CTRL (*P* = 0.028).

On the Zimbardo Time Perspective Inventory, no significant between-group differences were found (all *P* values > 0.192) (Present hedonistic: bvFTD = 3.4 ± 0.3, AD = 3.2 ± 0.6, CTRL = 3.5 ± 0.3; Present fatalistic: bvFTD = 2.7 ± 0.7, AD = 2.9 ± 0.5, CTRL = 2.4 ± 0.5; Future: bvFTD = 3.4 ± 0.4, AD = 3.7 ± 0.2, CTRL = 3.5 ± 0.4).

### Correlations

Correlations between delay discounting and judgement of valence and arousal were apparent only in AD, where decreased delay discounting in the positive condition correlated with increased judgement of positive valence (r_(10)_ = −0.802, *P* = 0.002; Figure 2E).

### Neuroimaging results

#### Patterns of atrophy

Patterns of atrophy in the clinical groups were typical of these diseases (Nestor *et al.*, 2003; Seeley *et al.*, 2008; Landin-Romero *et al.*, 2017) (Supplementary Figure 1; Supplementary Table 1). Compared with CTRL, bvFTD showed decreased grey matter intensity in the medial prefrontal cortex, frontal and temporal gyri, ACC, as well as subcortical regions including the hippocampus and striatum. In contrast, AD showed a significant bilateral decrease of grey matter intensity in the medial temporal lobe, including the hippocampus and amygdala, as well as in the precuneus and the insula.

#### Neural correlates of delay discounting

**Fig. 3:**
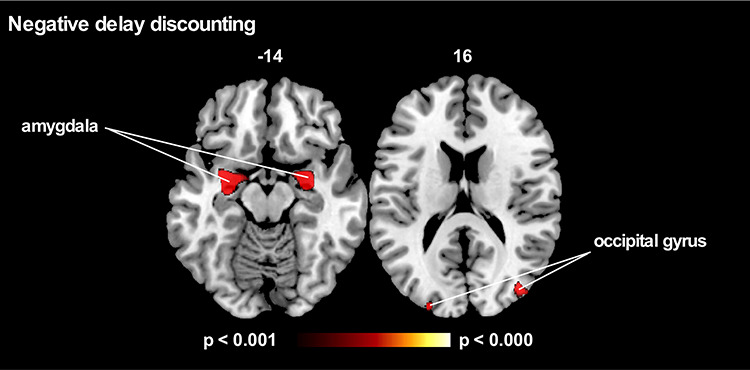
VBM analyses showing regions negatively correlated with delay discounting in the Negative condition irrespective of diagnosis. No clusters survived in the Positive or Neutral conditions (*P* < 0.001 uncorrected for multiple comparisons). Age and total intracranial volume included as a covariate in all VBM analyses. Clusters are overlaid on the standard MNI brain. The left side of the image is the left side of the brain.

Correlations between POS, NEG and NEU delay discounting and grey matter intensity revealed that, irrespective of diagnosis, increased delay discounting in the NEG condition was associated with reduced grey matter integrity in the amygdala (*P* < 0.001, cluster FWE-corrected) and occipital gyrus bilaterally (*P* < 0.001, uncorrected; Figure 3; Table 2). In contrast, no specific patterns of association emerged for the positive and neutral conditions.

**Table 2:** Clusters associated with greater delay discounting in the Positive, Negative and Neutral conditions across all three groups (bvFTD, AD and CTRL).

Regions	Laterality	MNI			Voxels
		x	y	z	
**Delay discounting: positive condition**					
No significant cluster identified					
**Delayed discounting: negative condition**					
Parahippocampal gyrus, amygdala and hippocampus	L	−31	−3	−17	3495
Parahippocampal gyrus, amygdala and hippocampus	R	31	−2	−17	2639
Middle occipital gyrus	R	38	−80	16	833
Middle occipital gyrus	L	−23	−93	21	462
Superior occipital cortex	R	21	−94	23	448
Precuneus	R	40	−78	38	276
Middle frontal gyrus	L	−28	40	40	198
Superior temporal lobe	L	−63	−9	3	191
Inferior frontal gyrus	R	51	20	20	141
Insula	L	−41	−7	−6	117
**Delayed discounting: neutral condition**					
No significant cluster identified					

**Fig. 4:**
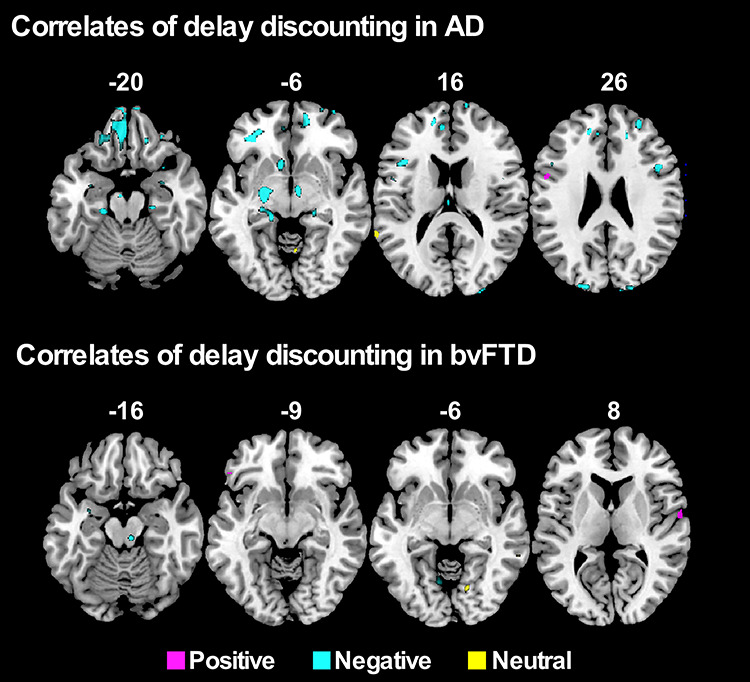
VBM analyses showing regions negatively correlated with delay discounting in AD and bvFTD in the Positive, Negative and Neutral conditions (*P* < 0.001 uncorrected for multiple comparisons). Age and total intracranial volume included as a covariate in all VBM analyses. Clusters are overlaid on the standard MNI brain. The left side of the image is the left side of the brain.

Further analyses on each patient group combined with controls showed distinct patterns of grey matter intensity in bvFTD and AD correlating with POS, NEG or NEU delay discounting (Figure 4; Table 3). Increased delay discounting in the NEG condition in AD was associated with reduced grey matter intensity in bilateral amygdala, vmPFC, ACC and hippocampus. No such associations were observed in the bvFTD group. Marginal frontal and temporal areas were associated with positive and neutral delay discounting, respectively, in AD and bvFTD.

**Table 3:** Clusters associated with greater delay discounting in the Positive, Negative and Neutral conditions for AD and bvFTD groups separately.

Regions	Laterality	MNI			Voxels
		x	y	z	
AD					
Positive delay discounting					
Inferior frontal gyrus	L	−53	4	23	382
Negative delay discounting					
Orbitofrontal frontal cortex	L/R	−35	37	−6	7861
ACC	L	−6	51	6	2035
Parahippocampal gyrus and hippocampus	L	−28	−24	−32	1809
Parahippocampal gyrus and hippocampus	R	25	−22	−29	1443
Amygdala	L	−24	−14	−7	1225
Middle and superior frontal gyrus	R	26	21	53	1074
Middle frontal gyrus	R	27	55	23	942
Inferior, middle and superior occipital gyrus	L	−20	−93	21	746
Supramarginal gyrus	L	−44	−39	32	803
Superior occipital cortex	R	21	−95	23	657
Inferior frontal gyrus	L	−40	17	16	463
Middle and superior frontal gyrus	L	−14	3	58	396
Inferior frontal gyrus	R	47	13	25	379
Amygdala	R	31	−1	−20	326
Inferior temporal gyrus	R	48	−18	−39	261
Neutral delay discounting					
Superior temporal gyrus	L	−66	−47	17	75
**bvFTD**					
**Positive delay discounting**					
Superior temporal gyrus	R	62	−6	8	135
Inferior frontal gyrus	L	−48	29	−13	87
**Negative delay discounting**					
Lingual gyrus	L	−9	−64	−4	247
Inferior parietal lobule	L	−61	−41	43	148
Supramarginal gyrus	L	−59	−55	36	103
**Neutral delay discounting**					
Rolandic operculum	R	15	−70	−5	186

## Discussion

This study revealed different patterns of modulation of emotion on decision-making in the two most common younger-onset dementia syndromes, AD and bvFTD, which were associated with specific neural changes. Supporting our hypotheses, bvFTD patients showed greater delay discounting compared to AD and controls, but no modulation according to emotion. In contrast, AD patients showed increased delay discounting in the negative condition, which was associated with greater bilateral amygdala atrophy. No specific pattern of brain atrophy was observed in bvFTD.

The increased impulsivity observed in bvFTD aligns with previous studies reporting impulsive decision-making in this population (Strenziok *et al.*, 2011; Gleichgerrcht *et al.*, 2012; Bertoux *et al.*, 2013, 2015b; Kloeters *et al.*, 2013; Lebreton *et al.*, 2013; Lansdall *et al.*, 2017; Beagle *et al.*, 2020). One recent report, however, failed to show any deficits on the delay discounting task in bvFTD compared with AD and controls (Chiong *et al.*, 2016). The authors argued that this was due to the very early disease stage of their patients. Our findings challenge this interpretation as we find evidence of decision-making deficits on the delay discounting task in patients with a similar disease severity (mean MMSE = 26, converted from ACE-III score, Matias-Guiu *et al.*, 2018).

As anticipated, compared to AD, bvFTD patients failed to show the negative emotion-induced modulation of delay discounting, a finding compatible with a primary deficit in emotion processing in bvFTD. Patients with bvFTD indeed show deficits in recognizing negative emotions (Lough *et al.*, 2006; Goodkind *et al.*, 2015) and emotional expression in faces and voices (Keane *et al.*, 2002; Lavenu and Pasquier, 2005) and emotional blunting (Mendez *et al.*, 2006). Grossmann et al. (2010) showed that bvFTD patients were also less sensitive to negative contextual features when making social decisions: negatively biased scenarios were judged as less negative than controls in bvFTD, whereas positively biased scenarios were rated equally in bvFTD and controls. Alternatively, these findings could be due a failure in decoding the physiological arousal signals in response to negative emotional stimuli. Indeed, previous studies have reported reduced physiological responses (e.g. skin conductance) in response to emotional videos (Kumfor *et al.*, 2019), unpleasant odours (Perry *et al.*, 2017) or pain (Fletcher *et al.*, 2015). bvFTD indeed judged pictures as less arousing than AD and controls, whereas valence ratings were similar across groups. This indicates that the emotional impairment in bvFTD results from a reduced arousal triggered by the pictures rather than a primary deficit in recognizing their emotional content. The specificity of the effect to the negative condition in AD could follow from an effect of arousal on delay discounting rather than an effect of negative emotion *per se*. Studies have indeed shown that arousing pictures, regardless of emotion, increased delay discounting compared to neutral pictures (Ariely and Loewenstein, 2006; Sohn *et al.*, 2015). Future studies using objectives measures of arousal (e.g. skin conductance) are needed to clarify this point.

Across groups, increased delay discounting for the negative (but not the positive or neutral) condition was associated with reduced grey matter intregrity in bilateral amygdala and occipital gyri. Group-specific analyses indicated that this association was mediated primarily by the AD group which showed reduced grey matter integrity in bilateral amygdala, vmPFC and parahippocampal gyri that correlated with increased delay discounting in the negative condition. These findings indicate that the amygdala is involved in delay discounting, especially within an emotionally negative context. Our findings demonstrate for the first time in humans that amygdala damage increases delay discounting, mirroring animal studies where excitotoxic lesions of the BLA increased delay discounting (Winstanley *et al.*, 2004; Floresco and Ghods-Sharifi, 2007; Ghods-Sharifi *et al.*, 2009). Impact of amygdalar damage on various decision-making tasks has been reported before (Bechara *et al.*, 1999; Bar-On *et al.*, 2003; Hanten *et al.*, 2006; Brand *et al.*, 2007; Weller *et al.*, 2007; De Martino *et al.*, 2010), but never on delay discounting to date.

The direction of the correlation between amygdala integrity and delay discounting was not anticipated given the known role of the amygdala in processing negative emotion. This finding adds to the structural neuroimaging controversy in the field of delay discounting as to whether delay discounting is correlated with increased or decreased grey matter intensity (Cho *et al.*, 2013; Tschernegg *et al.*, 2015; Pehlivanova *et al.*, 2018). Importantly, although central to negative emotion processing, the amygdala is not the only brain region supporting negative emotion processing. Indeed, lesion studies have shown that the amygdala is necessary but not sufficient to process negative emotions as, apart from fear, amygdala damage does not preclude from triggering and feeling other negative emotions (Anderson and Phelps, 2002; Feinstein *et al.*, 2011). One candidate region is the vmPFC, which regulates emotion through top-down inhibition of the amygdala (Andrewes and Jenkins, 2019). Deficient inhibitory control of the vmPFC on the amygdala has been shown to lead to hyper-emotional reactivity and pathologically elevated levels of negative affect (Quirk and Gehlert, 2003; Milad *et al.*, 2006; Rauch *et al.*, 2006; Motzkin *et al.*, 2015). In situations where the affective/emotional signals are absent (i.e. delay discounting with no emotional component or neutral emotion), the amygdala would be less involved, possibly favoring the vmPFC recruitment (Sellitto *et al.*, 2011; Peters and D'Esposito, 2016). This interpretation is consistent with our lack of amygdala involvement in the neutral delay discounting condition. Our study suggests that the amygdala is involved in delay discounting rather than purely in processing emotions, in line with animal studies (Winstanley *et al.*, 2004; Floresco and Ghods-Sharifi, 2007; Ghods-Sharifi *et al.*, 2009).

The association that we found between increased delay discounting in the negative condition and reduced grey matter intensity in the occipital cortex further demonstrates the involvement of broad network during emotion processing and delay discounting task. fMRI and lesion studies have shown that emotional stimuli, particularly arousing, negative, stimuli recruit not only the amygdala but also the visual cortices (Vuilleumier *et al.*, 2004; Sabatinelli *et al.*, 2009; Motzkin *et al.*, 2015). Similarly, involvement of the occipital cortex on delay discounting tasks has been attributed to visual attention (Luo *et al.*, 2009) or vividness of imagined event in episodic delay discounting tasks (Hu *et al.*, 2016) (Luo *et al.*, 2009; Olson *et al.*, 2009).

Some limitations should be acknowledged. Our sample of bvFTD patients was heterogenous in terms of disease severity, disease duration and atrophy pattern compared to AD, which may have precluded other correlations with other brain regions (e.g. vmPFC) to emerge in this group. It should be noted, however, that the absence of correlation in the bvFTD group alone does not indicate that both dementia groups statistically differed. Future studies using larger and more homogeneous groups are needed to resolve these concerns. Importantly, whereas the role of the vmPFC in delay discounting has been clearly demonstrated from lesion studies (Sellitto *et al.*, 2011; Peters and D'Esposito, 2016) and brain stimulation studies (Manuel *et al.*, 2019), evidence from bvFTD is less convincing (Chiong *et al.*, 2016) even in very impaired and homogeneous bvFTD samples. Nevertheless, this limitation does not detract from our main message demonstrating the role of the amygdala in emotional delay discounting.

The absence of the predicted pattern of increased delay discounting in the negative condition in healthy controls when all reward magnitudes were grouped was unexpected. It is likely that this lack of emotion-induced modulation of delay discounting follows from overall reduced variability and impulsivity in our healthy control group, which prevented emotion-related modulations to clearly emerge. Emotion-induced modulation of delay discounting may thus be apparent only under high impulsivity conditions. Supporting this interpretation, our findings show that older controls did exhibit the negative emotion-induced increase in delay discounting but only under the condition of highest impulsivity (i.e. low-magnitude trials). Effects of emotion on delay discounting have been typically reported in young healthy adults (Hirsh *et al.*, 2010; Augustine and Larsen, 2011; Benoit *et al.*, 2011; Liu *et al.*, 2013; Lin and Epstein, 2014; Luo *et al.*, 2014; Guan *et al.*, 2015; Sohn *et al.*, 2015; Zhang *et al.*, 2018). Findings on age-related differences on delay discounting have been mixed. Several studies have reported young individuals to be more impulsive on delay discounting tasks compared to older adults (Green *et al.*, 1999; Whelan and McHugh, 2009; Jimura *et al.*, 2011; Lockenhoff *et al.*, 2011; Eppinger *et al.*, 2012). Other studies, however, have shown no age-related differences (Samanez-Larkin *et al.*, 2011; Roalf *et al.*, 2012; Rieger and Mata, 2015; Seaman *et al.*, 2016) or even increased delay discounting with age (Read and Read, 2004). In sum, although reduced compared to what we could have expected in young individuals, the control group did show emotion-induced modulation of delay discounting. The bvFTD group in contrast showed no emotion-induced modulation of delay discounting for any reward magnitude, further supporting their deficit in emotion processing.

Altogether, this study demonstrates the close connections between emotion processing and decision-making and the conditions under which these vary, in this instance dementia. Our findings have relevance for policymakers when developing health warning messages that aim to dissuade risky behaviors (Nan and Qin, 2019) or for improving negative health behaviors associated with increased delay discounting in clinical populations. A recent study showed promising findings demonstrating that computerized working memory training decreases the rate of delay discounting in older controls (Felton *et al.*, 2019). Improvements in emotion recognition have been reported after computerized emotion recognition training in schizophrenia (Russell *et al.*, 2006) and Huntington’s disease (Kempnich *et al.*, 2017) suggesting an avenue for emotion recognition training as a mean of reducing impulsivity. These clinical interventions based on costs/benefits and emotion detection are however more likely to work in AD than in bvFTD.

## Funding

This work was supported by funding to ForeFront, a collaborative research group dedicated to the study of frontotemporal dementia and motor neuron disease, from the National Health and Medical Research Council (NHMRC) (APP1037746) and the Australian Research Council (ARC) Centre of Excellence in Cognition and its Disorders Memory Program (CE11000102). ALM is supported by the Swiss National Science Foundation, grant no. P300P1_171478 and P4P4PS_183817. OP is supported by an NHMRC Senior Research Fellowship (GNT1103258). RLR is supported by the Appenzeller Neuroscience Fellowship and the ARC Centre of Excellence in Cognition and its Disorders Memory Program (CE110001021). FK is supported by an NHMRC-ARC Dementia Research Development Fellowship (GNT1097026).

## Declarations of interest

None.

## Supplementary Material

scan-20-016-File007_nsaa085
