# Supplementary material for: Interactions between decision-making and emotion in behavioral-variant frontotemporal dementia and Alzheimer’s disease
Source: Soc Cogn Affect Neurosci. 2020 Jul 1;15(6):681–94. doi: 10.1093/scan/nsaa085 (PMC7393308; doi:10.1093/scan/nsaa085)
Supplement: scan-20-016-File007_nsaa085 [file scan-20-016-file007_nsaa085.docx]

**Supplementary material**


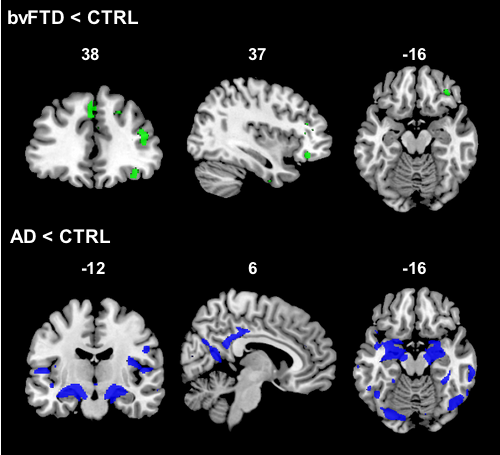


**Supplementary Figure 1**: Differences in grey matter intensity between patient groups and controls. Regions of grey matter intensity difference between bvFTD and controls (green) and AD and controls (blue; *p* < 0.001 uncorrected for multiple comparisons). Age and total intracranial volume included as a covariate in all VBM analyses. Clusters are overlaid on the standard MNI brain. The left side of the image is the left side of the brain.


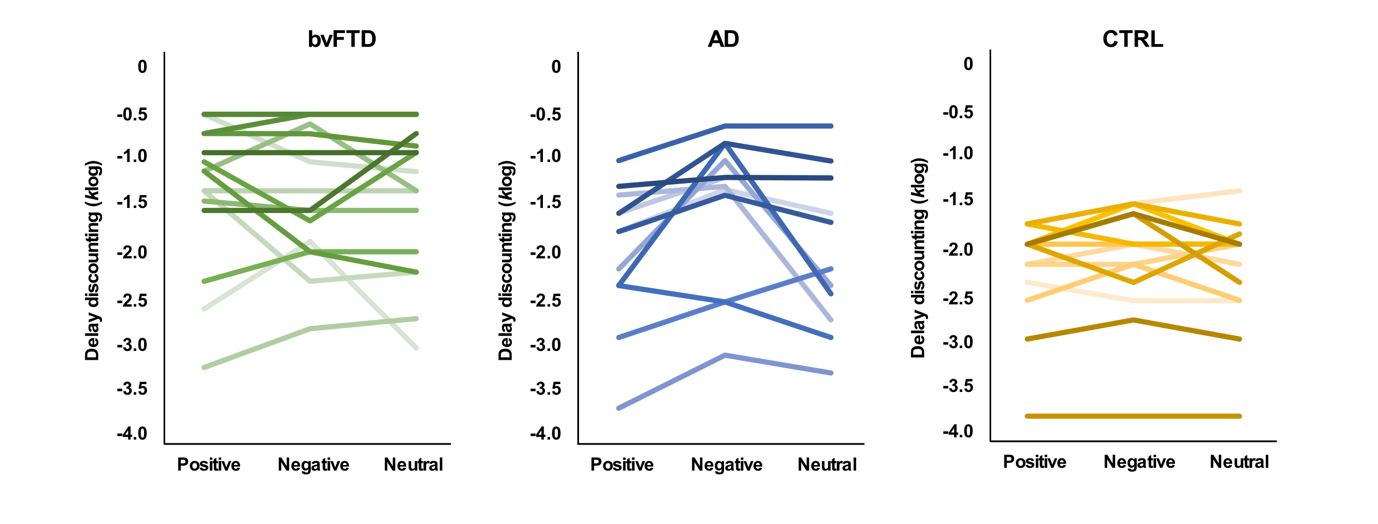


**Supplementary Figure 2:** Individual delay discounting rate for bvFTD, AD and controls. Shades of colour represent distinct individuals in each group (green: bvFTD; blue: AD; yellow: CTRL).

**Supplementary Table 1**: Group differences in grey matter integrity in patient groups compared to controls.

| **Regions** | **Laterality** |  | **MNI** |  | **Voxels** | |
| --- | --- | --- | --- | --- | --- | --- |
|  |  | **x** | **y** | **z** | |  |
| **bvFTD < Controls** |  |  |  |  |  | |
| Superior medial frontal gyrus, anterior cingulate cortex | L/R | -1 | 36 | 42 | 2492 | |
| Middle and inferior frontal gyrus | L/R | 44 | 40 | 17 | 1362 | |
| Inferior, middle and superior temporal gyrus extending into parahippocampal gyrus, hippocampus, caudate | L/R | -40 | -5 | -35 | 1070 | |
| Medial orbitofrontal cortex | R | 34 | 39 | -18 | 452 | |
| Postcentral gyrus, supramarginal gyrus, superior temporal gyrus | L/R | -59 | -22 | 18 | 337 | |
| Inferior occipital gyrus | L | -19 | -94 | -7 | 195 | |
| **AD < Controls** |  |  |  |  |  | |
| Middle temporal gyrus extending bilaterally into the amygdala, parahippocampal gyrus, hippocampus, Heschl gyrus, insula, precuneus, anterior cingulate cortex, inferior and superior parietal and occipital cortices | L/R | -18 | -2 | -14 | 329480 | |
| Postcentral gyrus, supramarginal gyrus, inferior parietal lobule | R | 52 | -24 | 43 | 17262 | |
| Inferior, middle and superior frontal gyrus | L | -19 | 36 | 39 | 13576 | |
| Thalamus | L | -7 | -18 | 17 | 1416 | |
| Inferior and medial frontal gyrus | R | 52 | 26 | -6 | 551 | |
| Caudate | R | 15 | 18 | 14 | 176 | |
